# Serotype-dependent adhesion of Shiga toxin-producing *Escherichia coli* to bovine milk fat globule membrane proteins

**DOI:** 10.3389/fmicb.2022.1010665

**Published:** 2022-11-24

**Authors:** Arthur Bagel, Christelle Lopez, Elisabeth David-Briand, Valérie Michel, Thomas Douëllou, Delphine Sergentet

**Affiliations:** ^1^Bacterial Opportunistic Pathogens and Environment Research Group, UMR5557 Ecologie Microbienne Lyon, National Center of Scientific Research (CNRS), Université de Lyon, Marcy-l’Etoile, France; ^2^INRAE, UR BIA, Nantes, France; ^3^Actalia, La Roche-sur-Foron, France; ^4^Laboratoire d’Etudes des Microorganismes Alimentaires Pathogènes, VetAgro Sup—Campus Vétérinaire, French National Reference Laboratory for Escherichia coli Including Shiga Toxin-Producing E. coli (NRL-STEC), Université de Lyon, Marcy-l‘Etoile, France

**Keywords:** food-borne pathogen bacteria, Shiga toxin-producing *Escherichia coli*, outer membrane proteins, raw milk, milk fat globule membrane, bacterial adhesion, food safety, anti-adhesion therapy

## Abstract

Shiga toxin-producing *Escherichia coli* (STEC) are food-borne pathogens that can cause severe symptoms for humans. Raw milk products are often incriminated as vehicule for human STEC infection. However, raw milk naturally contains molecules, such as the milk fat globule membrane and associated proteins, that could inhibit pathogen adhesion by acting as mimetic ligands. This study aimed to: (i) evaluate the capability of STEC cells to adhere to bovine milk fat globule membrane proteins (MFGMPs), (ii) highlight STEC surface proteins associated with adhesion and (iii) evaluate the variation between different STEC serotypes. We evaluated the physicochemical interactions between STEC and milk fat globules (MFGs) by analyzing hydrophobic properties and measuring the ζ-potential. We used a plate adhesion assay to assess adhesion between MFGMPs and 15 *Escherichia coli* strains belonging to three key serotypes (O157:H7, O26:H11, and O103:H2). A relative quantitative proteomic approach was conducted by mass spectrometry to identify STEC surface proteins that may be involved in STEC-MFG adhesion. The majority of *E. coli* strains showed a hydrophilic profile. The ζ-potential values were between −3.7 and − 2.9 mV for the strains and between −12.2 ± 0.14 mV for MFGs. Our results suggest that non-specific interactions are not strongly involved in STEC-MFG association and that molecular bonds could form between STEC and MFGs. Plate adhesion assays showed a weak adhesion of O157:H7 *E. coli* strains to MFGMPs. In contrast, O26:H11 and O103:H2 serotypes attached more to MFGMPs. Relative quantitative proteomic analysis showed that the O26:H11 str. 21,765 differentially expressed five outer membrane-associated proteins or lipoproteins compared with the O157:H7 str. EDL933. This analysis also found strain-specific differentially expressed proteins, including four O26:H11 str. 21,765-specific proteins/lipoproteins and eight O103:H2 str. PMK5-specific proteins. For the first time, we demonstrated STEC adhesion to MFGMPs and discovered a serotype effect. Several outer membrane proteins—OmpC and homologous proteins, intimin, Type 1 Fimbriae, and AIDA-I—that may be involved in STEC-MFG adhesion were highlighted. More research on STEC’s ability to adhere to MFGMs in diverse biological environments, such as raw milk cheeses and the human gastrointestinal tract, is needed to confirm the anti-adhesion properties of the STEC-MFG complex.

## Introduction

Shiga toxin-producing *Escherichia coli* (STEC) are food-borne pathogens whose source is most often the digestive tract of ruminants. Ingestion of ruminant-derived raw products, such as uncooked meat or raw milk dairy products, contaminated with STEC may result in a human infection. Water and vegetation soiled by ruminant feces are also incriminated as vehicule for human STEC infection (Kim et al., 2020). The Shiga toxins in STEC may cause mild symptoms or more severe conditions such as hemorrhagic colitis and hemolytic and uremic syndrome (HUS). STEC was the fourth most common zoonotic infection in the EU in 2020, after campylobacteriosis, salmonellosis, and yersiniosis (EFSA and ECDC, 2021). According to the latest WHO and FAO report, almost 2.5 million STEC cases occurred worldwide in 2010, resulting in 3,330 HUS cases, 200 cases of end-stage renal disease (ESRD), 27,000 Disability-Adjusted Life Years (DALY), and 269 deaths (FAO and WHO, 2018). Foodborne diseases are believed to account for half of the STEC disease burden.

These data suggest that both the risk of major sequelae after STEC infection and the case-fatality ratio are low, resulting in a modest illness burden at the population level. This, however, ignores the considerable cost placed on individual patients and their families, as well as the economic and trade implications of this food-borne pathogen. More than 470 STEC serotypes have been identified and almost 130 of these are associated with human health consequences (Zhang et al., 2022). The most common STEC serotype linked to foodborne outbreaks and human illnesses is STEC O157:H7. Other non-O157:H7 STEC serotypes have been a major source of foodborne outbreaks and sporadic infections in recent years (Valilis et al., 2018; The Joint FAO/WHO Expert Meetings on Microbiological Risk Assessment (JEMRA), 2019). STEC O26:H11 and O103:H2 serotypes are most commonly linked to raw dairy product-mediated STEC infection (Douëllou et al., 2016).

The ability to adhere to the intestinal epithelium and colonize the intestine undeniably contributes to the pathogenicity of STEC cells. A vast majority of the STEC clinical isolates known to cause bloody diarrhea or HUS have one or more virulence factors that allow their adhesion to intestinal epithelial cells (FAO and WHO, 2018). The major adhesion factor of clinical STEC isolates is intimin, a protein encoded by the *eae* gene that resides in the locus of an enterocyte effacement pathogenicity island (LEE) (The Joint FAO/WHO Expert Meetings on Microbiological Risk Assessment (JEMRA), 2019). To effectively colonize a host and cause disease, STEC has evolved mechanisms and strategies for attaching and adhering to host cells and tissues (Koutsoumanis et al., 2020). Adhesion prevents STEC cells from being swept away by the host’s natural self-cleaning mechanisms and, therefore, allows colonization and growth at a host-specific site (The Joint FAO/WHO Expert Meetings on Microbiological Risk Assessment (JEMRA), 2019). Thus, adhesion is a key step in the bacterial pathogenic mechanism, and inhibiting this early step may prevent infection.

Bacterial adhesion is a complex mechanism involving a non-specific and a specific phase. Non-specific interactions are the consequence of attractive and repulsive forces between the bacterium and the surface, allowing their rapprochement. These forces include all non-covalent interactions such as electrostatic interactions or surface charges, Van der Waals and Lewis acid/base interactions, as well as hydrophobic interactions (van Loosdrecht et al., 1987; Zita and Hermansson, 1997). Hydrophobic interactions and surface charges are the primary forces influencing bacterial adhesion (Berne et al., 2018). Hydrophobic interactions are defined as the ability of two components of similar hydrophobicity to attract each other (Krasowska and Sigler, 2014). Electrostatic forces result from the presence of a double ionic layer at the surface of a particle. If two membranes have opposing charges, they repel each other and, therefore, prevent progression to the second phase. The second bacterial adhesion phase, called the “specific phase,” involves molecular factors exposed on both target and bacterial cell surfaces. Adhesins are bacterial adhesion molecules that recognize oligosaccharide moieties or peptide residues on target cell surfaces. Porins, complex protein structures such as pili or flagella, glycoproteins, and glycolipids are all examples of adhesins.

Foods contain various ligands that could inhibit or limit pathogen adhesion to the intestinal epithelium. Raw milk contains free or protein-associated carbohydrates that could act as mimetic ligands for pathogens (Douëllou et al., 2017; Sun and Wu, 2017). Interestingly, the prevalence of STEC in raw milk products is high compared with the number of human infection cases, suggesting that the raw milk matrix can modulate the pathogenicity of STEC (Farrokh et al., 2013; Bagel and Sergentet, 2022). Full-fat raw milk cheeses can impair STEC adhesion to mouse enterocytes, while low-fat raw milk cheeses cannot (Douëllou et al., 2018). Furthermore, STEC can associate with milk fat globules (MFGs) in bovine raw milk and this affinity is serotype- and strain-dependent (Bagel et al., 2022). However, the underlying mechanisms of the STEC-MFG association remain unknown.

Milk fat globules are lipid droplets naturally secreted into milk by mammary epithelial cells (Lopez, 2020a) and comprise almost 98% of total raw milk fat (Lee et al., 2018). MFGs are composed of a triglyceride core surrounded by a biological membrane, called the milk fat globule membrane (MFGM), that is structured as a trilayer of polar lipids (phospholipids, sphingolipids, cholesterol) and membrane-specific proteins (glycoproteins, enzymes) (Lopez, 2020b). A rich diversity of proteins and glycoproteins, called the MFGM proteins (MFGMPs), as well as glycolipids, are anchored in the outer layer of the MFGM and could act as ligands for bacteria or viruses (Spitsberg, 2005; Douëllou et al., 2017). Interestingly, some MFGMPs have similarities with (glyco)-proteins of human intestinal cells, especially mucins that play a pivotal role in enterobacterial adhesion (Bagel and Sergentet, 2022).

In this study, we hypothesized that STEC could adhere to MFGs through molecular interactions between STEC and MFGMPs. The study aimed to evaluate the impact of serotype on STEC’s ability to adhere to bovine MFGMPs and to elucidate which STEC surface proteins are associated with an adhesion profile.

## Materials and methods

### Bacterial strains and culture conditions

Details of *E. coli* strains used in the following experiments are listed in Table 1. Bacteria were plated from glycerin-BHI frozen stock (−80°C) onto Luria-Bertani agar (LB) plates (Oxoïd, Thermo Fisher Diagnostics, Dardilly, France) and incubated for 24 h at 37°C. The day before the experiments, one colony was picked from the agar plate and cultured overnight at 37°C in Brain heart infusion (BHI) (BioMérieux, Marcy-1’Etoile, France). This procedure was performed for each experiment described below. The term “*E. coli”* refers to the entire collection.

**Table 1:** Collection of 16 *E. coli* strains used in the study, included O157 and non-O157 strains: O157:H7 (*n* = 5), O26:H11 (*n* = 5), O103:H2 (*n* = 5), and the non-pathogenic *E. coli* K-12 str. MG1655.

Strain	Intimin and Shiga-toxin genotype	Origin	Detailed origin	Isolation date	Reference
Serotype O157:H7					
SAKAI	eae*^+^ stx1^+^stx2^+^*	Human	HUS	1996	Hayashi et al. (2001)
EDL933	eae*^+^ stx1^+^ stx2^+^*	Human	HUS	1983	Perna et al. (2001)
1044	eae*^+^ stx1^−^ stx2^+^*	Dairy product	Bovine raw milk cheese	2012	a
5280-B	eae*^+^ stx1^+^ stx2^+^*	Dairy product	Bovine raw milk cheese	2019	a
2044-A	eae*^+^ stx1^−^ stx2^−^*	Dairy product	Bovine raw milk cheese	2016	a
Serotype O26:H11					
21765	eae*^+^ stx1^−^ stx2^+^*	Human	HUS	2005	Galia et al. (2015)
11368	eae*^+^ stx1^+^ stx2^−^*	Human	HUS	2001	Ogura et al. (2007)
103	eae*^+^ stx1^+^ stx2^−^*	Dairy product	HUS	2012	a
2157-A	eae*^+^ stx1^−^ stx2^+^*	Dairy product	Bovine raw milk cheese	2018	a
4315-A	eae*^+^ stx1^−^ stx2^−^*	Dairy product	Bovine raw milk cheese	2019	a
Serotype O103:H2					
PMK5	eae*^+^ stx1^+^ stx2^−^*	Human	HUS	1993	Mariani-Kurkdjian et al. (1993)
32396	eae*^+^ stx1^+^ stx2^+^*	Human	HUS	2011	b
1487-A	eae*^+^ stx1^+^ stx2^+^*	Dairy product	Bovine raw milk cheese	2018	a
2503	eae*^+^ stx1^+^ stx2^−^*	Dairy product	Bovine raw milk cheese	2012	a
445–14	eae*^+^ stx1^−^ stx2^−^*	Dairy product	Bovine raw milk cheese	2012	a
MG1655 K12	eae*^−^ stx1^−^ stx2^−^*	Human	Stool sample	1922	Bachmann (1996)

We paid close attention to the strains’ origin and virulence genes. For each sub-serotype, we retained two strains isolated from human cases and three others from cheese made from cow’s raw milk. In addition, we chose strains based on whether stx1 and/or stx2 genes were present (*n* = 4) or not (*n* = 1).

HUS, hemolytic and uremic syndrome.

a, French National STEC Reference Laboratory (VetAgro Sup, Marcy l’Etoile, France).

b, Mother and Child Hospital (Hospices Civils de Lyon, Bron, France).This E. coli strain collection was used in a previous study (Bagel et al., 2022).

### Solutions and products

Phosphate buffer (PB) was composed of NaH_2_PO_4_ and Na_2_HPO_4_ at 19.0 mmol.L^−1^ and 23.9 mmol.L^−1^, respectively, at pH = 6.8 and ionic strength = 80 mM. The washing/blocking buffer (WBB) was obtained by adding 5% Tween 20 to PB. The coating buffer (CB) was composed of Tris 63 mmol.L^−1^_,_ 2% SDS, 4% β-mercaptoethanol, and 20% glycerol. The pH was adjusted to 6.8 with a hydrochloric acid solution. All products were purchased from Sigma-Aldrich (Saint-Quentin-Fallavier, France).

### Raw milk, milk fat globules, and milk fat globule membrane proteins

Raw whole bovine milks were collected after the morning milking (7 a.m.) and provided by Gaec des Fougères (35 La Chapelle des Fougeretz, France).

MFGs were isolated from the milk aqueous phase to remove proteins (caseins, whey proteins) using a method adapted from The Joint FAO/WHO Expert Meetings on Microbiological Risk Assessment (JEMRA) (2019). Briefly, (i) the milk was warmed to 50°C for 10 min; (ii) 15 mL of raw whole milk were deposited, using a syringe, at the bottom of 50 mL plastic centrifuge tubes containing 30 ml of ultrafiltration milk permeate (UFMP; aqueous phase of milk with proteins and lipids removed), (iii) the tubes were centrifuged at 1600 *g* for 20 min in order to form a layer of washed MFGs at the top of the tubes that was collected for further investigations.

MFGMPs were obtained using a method developed in the INRAE lab: (i) raw whole bovine milks (4%wt fat) were centrifuged using a disc stack centrifuge (Elecrem) to obtain a cream (approximately 40%wt fat), (ii) the cream was dispersed in warmed water to reach 4%wt fat and centrifuged to remove the proteins (caseins, whey proteins), lactose and minerals (step repeated twice); (iii) the cream containing washed MFGs was stored at −20°C until further use; (iv) the cream was churned at 8°C using a household mixer (Kenwood) at speed 5 for 30 min; (v) the aqueous phase, called buttermilk and enriched in MFGM fragments, was obtained, filtered, and centrifuged at 12,000 *g* for 30 min; (vi) to remove the lipids, the MFGM-enriched pellet was treated with a mixture of ether/ethanol (1/3, v/v), shaken, stored one night at 20°C, and centrifuged at 1,200 *g* for 10 min at 10°C; (vii) the pellet was washed twice using the ether/ethanol mixture; (viii) the recovered MFGM proteins were dried using nitrogen and stored at 4°C in ethanol until futher utilization, (ix) SDS-PAGE confirmed the removing of milk proteins (caseins, whey proteins) and the presence of MFGMPs in the extract.

### Evaluation of the non-specific interactions between STEC and MFGs

#### *Escherichia coli* cell surface hydrophobicity

Cell surface hydrophobicity (CSH) of STEC strains was evaluated through the microbial adhesion to hydrocarbon (MATH) test (Rosenberg et al., 1980). For each strain, three independent stationary bacterial cultures were washed once in PB and calibrated at 5.10^8^ CFU.mL^−1^ in the same buffer. Then 300 μL of n-hexadecane (Fisher Scientific, Thermo Fisher Scientific, Lissieux, France) were added to 3 ml of bacterial suspension in a round bottom glass tube (1:10). Glass tubes were carefully placed in a densitometer (BioMérieux, Marcy-l’Etoile, France) to measure the initial bacterial turbidity (McF_O_). Then, glass tubes were vortexed for 2 min at maximal speed and set aside for 20 min to allow phase separation. The final turbidity (McF_20_) was measured as previously described. Cell surface hydrophobicity (CSH) was defined as the percentage of *E. coli* present in the hydrocarbon phase at the end of the experiment and was calculated by the following formula:


$$CSH\;\left(\%\right)=100\times \frac{{\mathrm{McF}}_{0}-{\mathrm{McF}}_{20}\;}{{\mathrm{McF}}_{0}}$$

#### *Escherichia coli* surface charge: ζ-potential measurements

The ζ-potential values of MFGs and AEEC strains were determined. An overnight bacterial culture (BHI; 37°C) of each AEEC strain, belonging to the three *E. coli* serotypes studied, was washed and calibrated at 9 log_10_ CFU.mL^−1^ in UFMP. The bacterial concentration was set according to the number of MFGs in raw milk, which is close to 9 log_10_ MFGs/mL of raw milk. Samples were then prepared by suspending 5–10 μL of raw whole bovine milk, washed MFGs, or calibrated bacterial cultures in 10 mL of UFMP to have the same pH and ionic strength as milk. The samples were added to a cuvette (1 mL), which was then placed into the chamber of a Zetasizer Nano ZS (Malvern, Germany). The ζ-potential was calculated from the electrophoretic mobility according to the Smoluchowski approximation and Henry’s law. The measurements were run five times at 25°C.

### Evaluation of the capacity of *Escherichia coli* to adhere to MFGMPs

**Figure 1:**
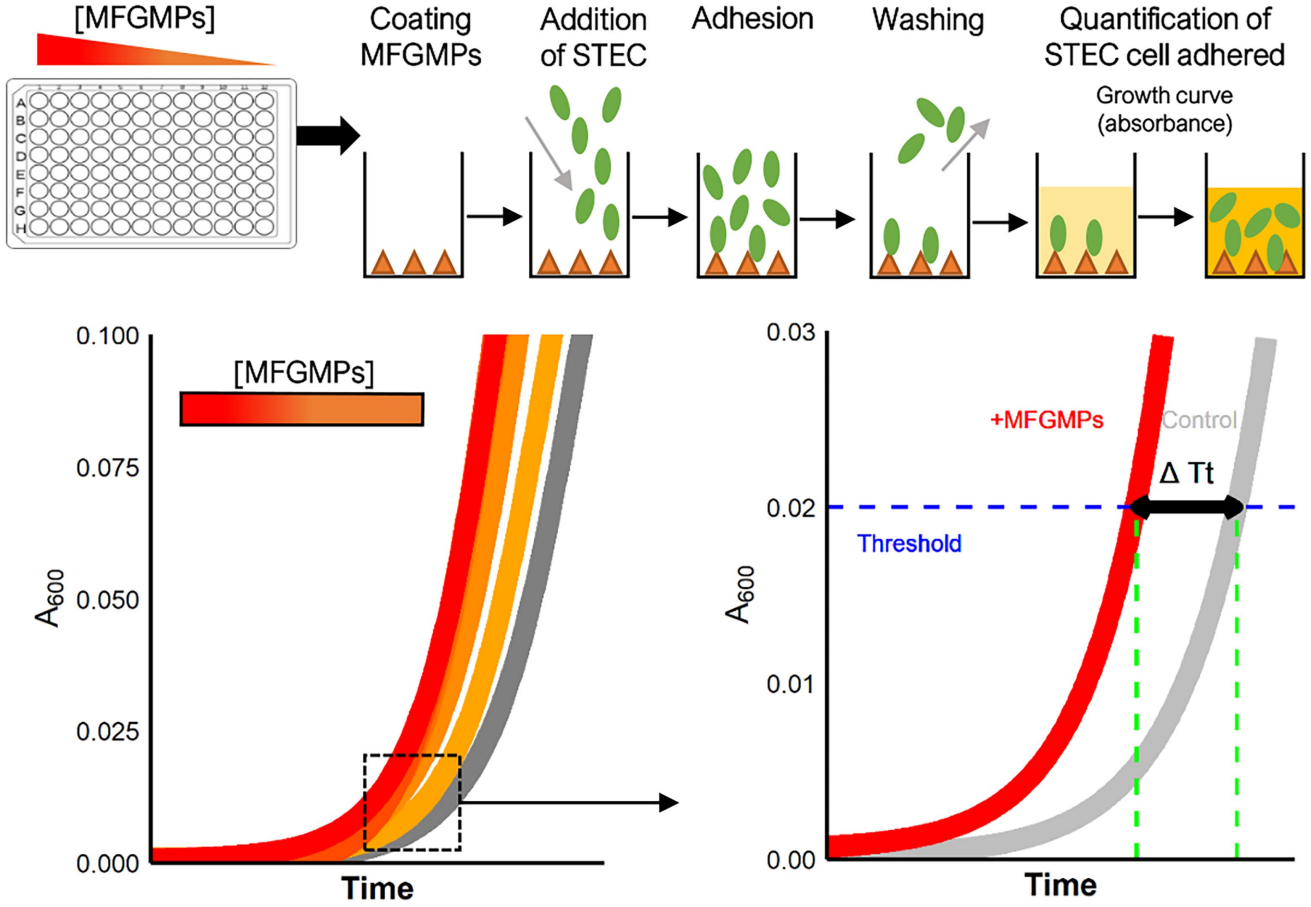
Experimental process for the plate adhesion assay. The assay was performed in sterilized 96-well plates coated with MFGMP solutions at various concentrations. *E. coli* cells were added to wells for 2 h at 4°C and the plates were extensively washed to remove non-adhering cells. Growth curves were acquired in a plate reader and a five-parameter log-logistic model was used to estimate the time of each curve to reach an absorbance of 0.02 at 600 nm (Time Threshold; Tt). The greater the number of bacterial cells adhered to the well, the shorter the time to reach the threshold. The difference in Tt (ΔTt in sec) with and without MFGMPs was calculated for each MFGMP coating solution, strain, and experimental replicate.

A plate adhesion assay was performed to evaluate the capacity of *E. coli* to adhere to MFGMPs. Figure 1 shows the main steps of the experiment. Dried-MFGM proteins were resuspended in coating buffer (CB), filtered at 0.22 μm with cellulose acetate microfiltration tubes (Clearspin), aliquoted, and stored at −80°C. For each assay, an aliquot of MFGMPs was thawed and four-fold dilutions were performed in CB. A 96-well plate (Nunc MaxiSorp plate, ThermoFisher, Dardilly) was UV-sterilized for 30 min and wells were coated overnight at 20 ± 1°C with 100 μL of MFGMP solution at 100, 25, 6.25, 1.56, or 0.39 μg.mL^−1^. Wells were also coated with MFGMP-free coating buffer as a control (basal level of adhesion). Coating solutions were removed and wells were washed three times with 250 μL of washing/blocking buffer. Wells were then blocked for 2 h at 20 ± 1°C with 250 μL of washing/blocking buffer. For each strain assayed, an overnight bacterial culture in stationary phase (BHI; 37°C) was washed in PB, calibrated at 8 log_10_ CFU.mL^−1^ (according to the OD_600nm_/CFU.mL^−1^ relation), and stored at 4°C for 2 h before being added to wells. The blocking buffer was removed and replaced with 100 μL of bacterial solution. The adhesion assays were performed at 4°C for 2 h in static conditions. Wells were washed five times with 250 μL of washing/blocking buffer to remove non-adherent bacteria. Wells were air-dried (5–10 min) to remove any traces of buffer. Washing and blocking steps were realized with Wellwash™ Microplate Washer (ThermoFisher). The number of adherent *E. coli* cells was estimated by the delay of growth. Bacterial growth was initiated by adding 100 μL of LB medium to each well. Plates were incubated in a plate reader (Spark, Tecan, Tecan France, Lyon) inside a large humidity cassette at 37°C. The absorbance at 600 nm was measured every 15 min, after a shaking step (15 s at 200 rpm), for 15 h. The number of adherent *E. coli* cells was estimated by the delay of the bacterial lag phase which is proportionnal to the number of bacterial cells (Hazan et al., 2012). This process was performed three times for each strain with independent bacterial overnight cultures. To limit plate edge effects, edge wells were not used.

For each growth curve, a five-parameter log-logistic fit was used to estimate the time (in seconds) to reach a 0.02 absorbance threshold (Tt; Time Threshold) as previously described (Hoffmann et al., 2018). The “R” (R Core Team, 2021) script provided by the authors was used and slightly adapted to our design plan. Subsequently, for each condition, we calculated the difference in Tt with and without MFGMPs (ΔTt) for each 96-well plate. An increase in adherent bacterial cells in a well was shown by ΔTt > 0 s, while a ΔTt < 0 s meant a loss of bacterial cells. The results of this experiment were expressed as the median of ΔTt from three independent repetitions and the standard deviation in seconds (median ± standard deviation). The linearity between the response variable (ΔTt in sec) and the explanatory variable (concentration of MFGMPs in μg.mL^−1^) after log_10_ transformation was evaluated by simple linear regression, according to serotype. For these calculations, “R” software and the “stats” package (R Core Team, 2021) were used. The independence, normal distribution, and homogeneity of the residuals of each linear regression were checked graphically and complementary statistical tests were used if necessary.

### Characterization of STEC surface proteins

#### STEC surface protein extraction

The STEC surface protein extraction protocol was adapted from (Del Canto et al., 2012). For each serotype studied, one strain was selected: O157:H7 str. EDL933; O26:H11 str. 21765; and O103:H2 str. PMK5. For each strain, three individual colonies were grown overnight in 10 ml BHI at 37°C. Stationary cultures were centrifuged at 5,000 g for 5 min, washed twice in PBS (v/v), re-suspended in 100 μL of PBS, and heated at 60°C for 30 min. Heated suspensions were centrifuged at 5,000 g for 5 min. The supernatants, which contained the extracted proteins, were filtered through a 0.22 μm cellulose acetate microfiltration tube at 13,000 g for 3 min to eliminate any remaining bacterial cells. Protein extractions were confirmed by protein and DNA quantification and SDS-PAGE electrophoresis. Nanoscale liquid chromatography coupled to tandem mass spectrometry (nanoLC-MS/MS) was used for quantitative and qualitative analysis.

#### Tandem mass tag quantitative proteomics

The Tandem Mass Tag (TMT) quantitative method was used for relative quantification of STEC surface proteins (Dayon et al., 2008). Approximately 50 μg of each surface protein extract was DNAse-treated (Turbo DNAse, ThermoFisher, Dardilly, France) at 37°C for 1 h and then prepared for mass spectrometry (MS) analysis using the easyPep kit (ThermoFisher) according to the manufacturer’s instructions. Samples were reduced and alkylated for 10 min at 95°C and then digested with LysC/Trypsin at a 1:10 ratio for 3 h at 37°C. Peptide samples were then purified on easyPep kit spin columns, dried, re-suspended in 50 μL of 100 mM tetraethylammonium bicarbonate (TEAB), and labeled according to the protocol supplied with the TMT™ 10plex 0.2 mg Kit (Thermo Scientific). Two micrograms of each labeled sample were pooled to ultimately have approximately 18 μg of protein. The pool was then desalted on a C18 spin column (Thermo Scientific Pierce). The samples were analyzed on a high-resolution orbitrap mass spectrometer Q Exactive HF (Thermo Scientific), coupled to nanoUHPLC (Thermo Scientific). Samples were analyzed in triplicate. One microliter of each sample was loaded onto a C18 Acclaim PepMap100 trap-column 300 μm ID x 5 mm, 5 μm, 100 Å (Thermo Fisher Scientific) for 3 min at 20 μL/min with 2% ACN, 0.05% TFA in H_2_O and then separated on a C18 Acclaim Pepmap100 nano-column, 50 cm x 75 μm i.d, 2 μm, 100 Å (Thermo Fisher Scientific) with a 100-min linear gradient from 3.2 to 20% buffer B (A: 0.1% FA in H2O, B: 0.1% FA in ACN), from 20 to 32% of B in 20 min and then from 32 to 90% of B in 2 min, hold for 10 min, and returned to the initial conditions in 1 min for 14 min. The total duration was set to 150 min with a flow rate of 300 nl/min and the oven temperature was kept constant at 40°C.

Labeled peptides were analyzed with the TOP15 HCD method: MS data were acquired in a data-dependent strategy selecting the fragmentation events based on the 15 most abundant precursor ions in the survey scan (375–1800 Th). The resolution of the survey scan was 120,000 at m/z 200 Th and for the MS/MS scan, the resolution was set to 45,000 at m/z 200 Th. The Ion Target Value for the survey scans in the Orbitrap and the MS/MS scan were set to 3E6 and 1E5, respectively, and the maximum injection time was set to 50 ms for the MS scan and 120 ms for the MS/MS scan. Parameters for acquiring HCD MS/MS spectra were as follows: collision energy = 33 and an isolation width of 0.7 m/z. The precursors with unknown charge state or a charge state of 1 and 8 or greater than 8 were excluded. Peptides selected for MS/MS acquisition were then placed on an exclusion list for 30 s using the dynamic exclusion mode to limit duplicate spectra. MS data were reprocessed with Proteome Discoverer 2.4 software (Thermo Scientific) with the Sequest HT search engine against the total UniProt *E. coli* database (862,106 entries; octobre 2021) and the addition of a contaminant database, filtered at a false positive rate of 1%. Precursor mass tolerance was set at 10 ppm, fragment mass tolerance was set at 0.02 Da, and up to two missed cleavages were allowed. Oxidation (M) and acetylation (Protein N-terminus) were set as variable modifications and TMT labeled peptides in primary amino groups (K and N-ter) and carbamidomethylation (C) were set as fixed modifications. Validation of identified peptides and proteins was done using a target decoy approach with a false positive (FDR < 1%) *via* Percolator. Protein quantitation was performed with the reporter ions quantifier node in Proteome Discoverer 2.4 software with an integration tolerance of 20 ppm and peptide and protein quantitation based on pairwise ratios and t-test. The ratios of each protein identified were calculated between the three strains.

The differentially expressed proteins (DEPs) in strains O26:H11 21,765 and O103:H2 PMK5, compared with strain O157:H7 EDL933, were extracted based on the values of a Log2 abundance ratio < −1 and a value of *p* <0.05. Further analysis or graphical representations were performed in “R” (R Core Team, 2021). Venn diagram and Gene Ontology (GO) term analysis were realized with “ggvenn” (Yan, 2022) and “UniprotR” (Soudy et al., 2020) packages, respectively. If the UniProt accession number assigned to a DEP was not fully annotated, the information was retrieved from homologous proteins in the UniProtKB database (>90% identity). DEPs associated with the outer membrane were selected from GO terms based on the presence of the keywords “cell adhesion” (biological process; BP) and/or “cell outer membrane” (cellular component; CC) and not located in the inner leaflet of the OM.

#### Qualitative proteomics

The equivalent of 2 μg of each TMT-labeled sample was mixed with the two other samples of the same strain, dried, re-suspended in 300 μL 0.1% trifluoroacetic acid (TFA), and finally desalted on a high-capacity spin column (Thermo Fisher Scientific). The samples were re-suspended in 10 μL 0.1% AF and 1 μL was analyzed with the same nanoLC method as previously described and a TOP20 DDA HCD mode: MS data were acquired in a data-dependent strategy selecting the fragmentation events based on the 20 most abundant precursor ions in the survey scan (375–1,500 Th). The resolution of the survey scan was 60,000 at m/z 200 Th and for the MS/MS scan, the resolution was set to 15,000 at m/z 200 Th. The Ion Target Value for the survey scans in the Orbitrap and the MS/MS scan were set to 3E6 and 1E5, respectively, and the maximum injection time was set to 60 ms for the MS and MS/MS scans. Parameters for acquiring HCD MS/MS spectra were as follows: collision energy = 27 and an isolation width of 2 m/z. The precursors with unknown charge state, charge state of 1 and 8, or greater than 8 were excluded. Peptides selected for MS/MS acquisition were then placed on an exclusion list for 20 s using the dynamic exclusion mode to limit duplicate spectra. MS data were reprocessed with Proteome Discoverer 2.5 software with the same parameters as previously described without reporter ion quantifier node. The initial database, composed of the total UniProt *E. coli* database (862,106 entries; octobre 2021) and a contaminant database, was completed with proteomic sequences of the three STEC strains obtained from genomic sequences *via* NCBI Prokaryotic Genome Annotation Pipeline (PGAP) (EDL933: 5425 entries; 21,765: 5361 entries; PMK5: 1994 entries).

The subcellular localization of identified proteins was predicted using the PSORTb 3.0 Subcellular Localization Prediction Tool (Yu et al., 2010). Spectral counts (SpC) of each protein were used to estimate protein abundance. Protein abundance was expressed through the normalized spectral abundance factor (NSAF). NSAF was calculated, for each strain, according to the formula:


$$NSAF=\frac{SAF_{i}}{\sum_{i=1}^{N}SAF_{i}}$$

where i is an unique protein and N is the total number of proteins, while SAF is a protein spectral abundance factor that is defined as a protein spectral count divided by its length, as described by Pisanu et al. (2011).

The NSAF was calculated only on proteins that had at least three peptide matches and at least three unique peptides. For each strain, the relative abundance of the subcellular localization groups was obtained by normalizing relative abundance to the number of proteins assigned in the corresponding group.

## Results

### Evaluation of the non-specific interactions between STEC and MFGs

**Figure 2:**
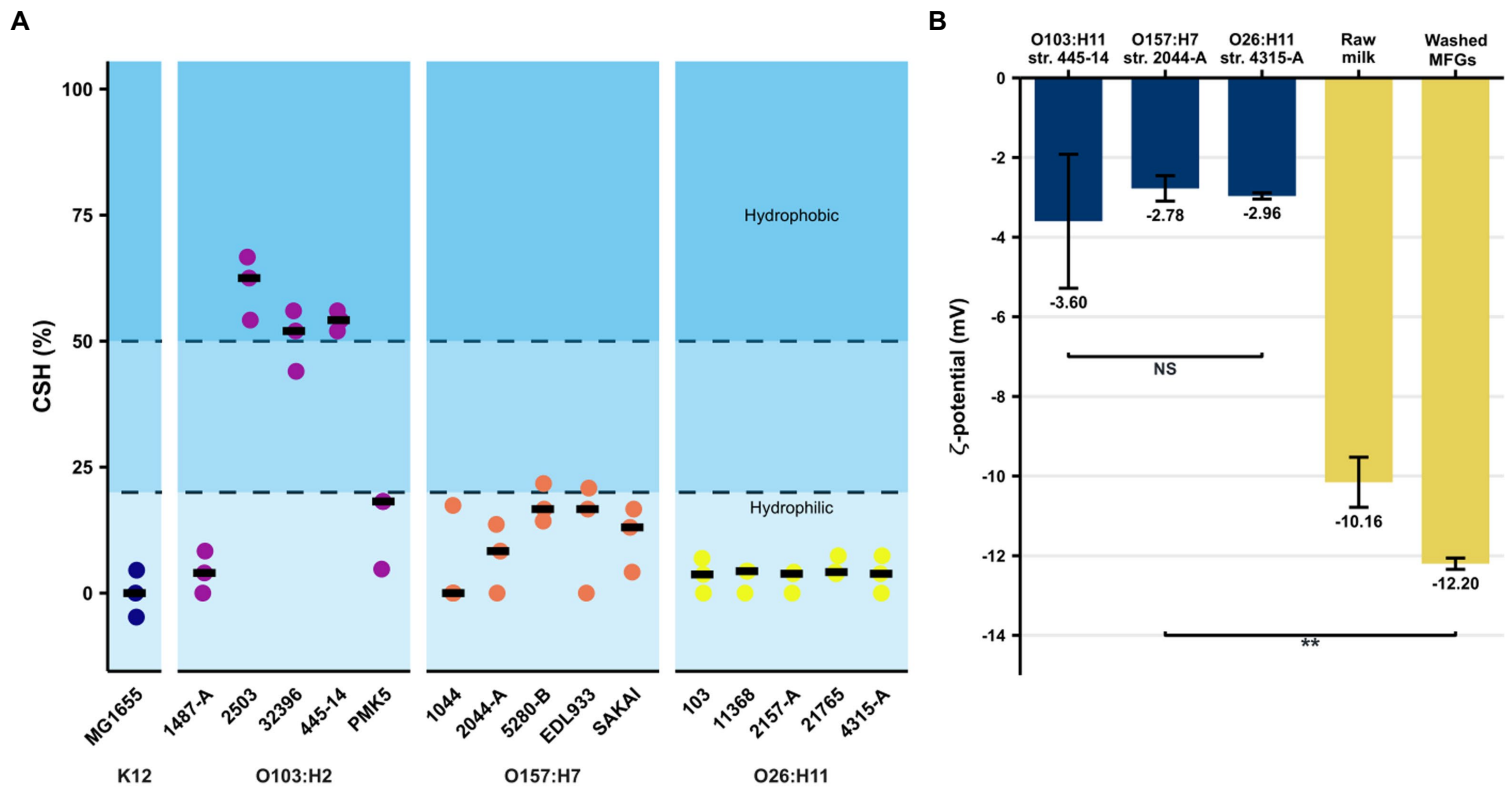
Evaluation of non-specific interactions between *E. coli* strains and bovine raw MFGs. **(A)** Cell surface hydrophobicity (in %) of the STEC strain collection evaluated by MATH test. A CSH ≤ 20% means a hydrophilic tendency while a value between 20 and 50% corresponds to moderately hydrophobic strains, and a result above 50% indicates a hydrophobic profile. Black bars represent the median of three independent experiments. **(B)** Measures of ζ-potential of MFGs in milk or dispersed in UFMP as well as the AEEC strains belonging to three key serotypes (O157:H7, O26:H11, and O103:H2). Bars correspond to the median and error bars represent the standard deviation. ** indicates significant differences (value of *p* <0.01), using the Wilcoxon signed-rank test. NS, not significant.

A value of CSH ≤ 20% signified a hydrophilic tendency, a value between 20 and 50% corresponded to moderately hydrophobic strains, and a result above 50% indicated a hydrophobic profile (Benito et al., 1997; Lamari et al., 2018). The majority of the STEC/AEEC strains assayed had a CSH < 25% and, therefore, a hydrophilic profile (Figure 2A). Three strains belonging to the O103:H2 serotype (str. 2,503, str. 32,396, str. 445–14) were hydrophobic, reaching a CSH between 45 and 70%. The *E. coli* K-12 MG1655 strain also showed a hydrophilic profile. The ζ-potential of MFGs in raw milk and in UFMP were − 10.2 ± 0.6 mV and − 12.2 ± 0.1 mV, respectively (Figure 2B). The three AEEC strains assayed were negatively charged and their ζ-potential were of the same order of magnitude, from −3.6 to −2.8 mV (Figure 2B).

### Evaluation of the capacity of *Escherichia* to adhere to milk fat globule membrane proteins

**Figure 3:**
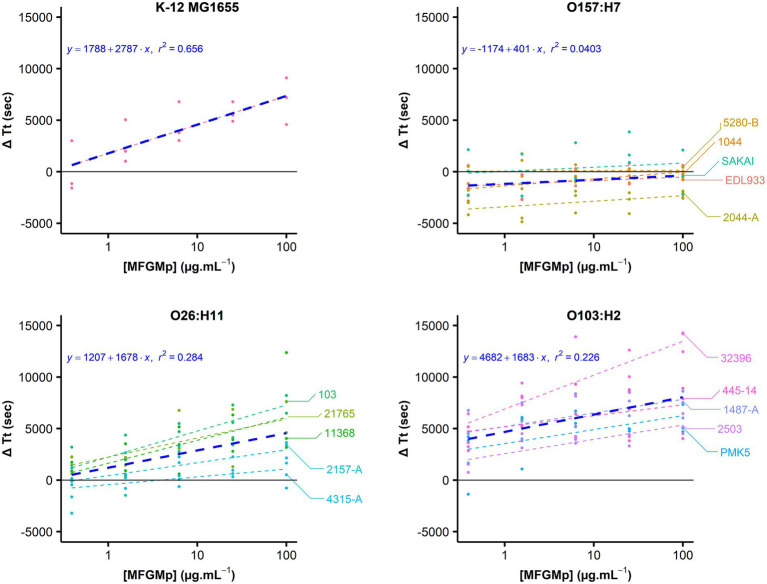
STEC adhesion to MFGMPs. The adhesion of *E. coli* to MFGMPs was evaluated by plate washing assay. Bacterial cells were added to wells previously coated with 100, 25, 6.25, 1.56, 0.39, or 0 μg.mL^−1^ MFGMPs at 4°C for 2 h. After extensive washing, growth curves were acquired in a plate reader and the time of each curve to reach an absorbance of 0.02 at 600 nm (Time Threshold; Tt) was estimated by a five-parameter log-logistic. The points represent the difference of Tt (ΔTt in sec) with and without MFGMPs. It was calculated for each coating solution, strain, and experimental replicate. For each strain, a dashed-line was plotted as a function of the median ΔTt for each MFGMP concentration (Log_10_ scale). Each strain is represented by a distinct color. The blue line represents the median of ΔTt for each MFGMP concentration at the serotype level. Three independent overnight bacterial cultures were used for each strain and assayed on different plates/days. The median and the standard deviations were calculated.

The increase in ΔTt as a function of the concentration of the MFGMP coating solution was graphically observed for the strains belonging to the O103:H2 and O26:H11 serotypes, as well as for the non-pathogenic K-12 MG1655 strain (Figure 3). This tendency was supported for these strains by the estimation of the slope of the regression equation’s line (value of *p* is 0.05; Table 2). At the serotype level, *E. coli* O103:H2 strains showed a ΔTt of 7,342 ± 3,391 s at 100 μg.mL^−1^ of MFGMPs, similar to the K-12 MG1655 strain (7,203 ± 2,276 s). As for the O26:H11 strains, a ΔTt of 3,846 ± 3,508 s was observed. However, at the strain level, the O26:H11 str. 4,315-A reached a ΔTt of 529 ± 1,460 s and, therefore, exhibited weak adhesion to MFGMPs. On the contrary, no concentration effect was demonstrated for the O157:H7 strains since the slope of the regression line was not significantly different from zero (value of *p* = 0.0841; Table 2). At the maximal MFGMP concentration assayed (100 μg.mL^−1^), the estimated ΔTt was −523 ± 1,347 s, suggesting a low capability to adhere to MFGMPs across all strains. At the maximal MFGMP concentration, a negative value for the delay of growth was estimated for all strains except for the 5,280-B strain. The results were − 420 ± 2,169, −785 ± 1,605, −2054 ± 311, −243 ± 415, and 425 ± 565, for the SAKAI, EDL933, 2044-A, 1044, and 5,280-B strains, respectively. Nevertheless, the O157:H7 str. 5,280-B displayed a negative ΔTt in some replicates (ΔTt_5280-B_ = [−140; 990 s]).

**Table 2:** Estimation of the coefficients and R^2^ of the linear regressions of STEC adhesion to MFGMPs.

	K-12 MG1655	O157:H7	O26:H11	O103:H2
Slope (ΔTt in sec)	2,786.896^***^ (560.140)	400.708^*^ (228.816)	1,678.107^***^ (318.459)	1,683.327^***^ (364.311)
Intercept (MFGMPs in μg.mL^−1^)	1,787.639^**^ (652.840)	–1,173.620^***^ (266.684)	1,206.764^***^ (366.363)	4,681.656^***^ (424.603)
*R* ^2^	0.656	0.040	0.284	0.226

For each serotype (O157:H7, O26:H11, O103:H2) and the K-12 MG1655 strain, a simple linear regression was used to assess the relationship between the time threshold for bacterial growth (ΔTt) and the amount of MFGMPs. Standard errors are indicated in parentheses.

*p < 0.1; **p < 0.05; ***p < 0.01.

### Characterization of STEC surface proteins

#### Qualitative proteomics analysis and relative protein abundance

**Figure 4:**
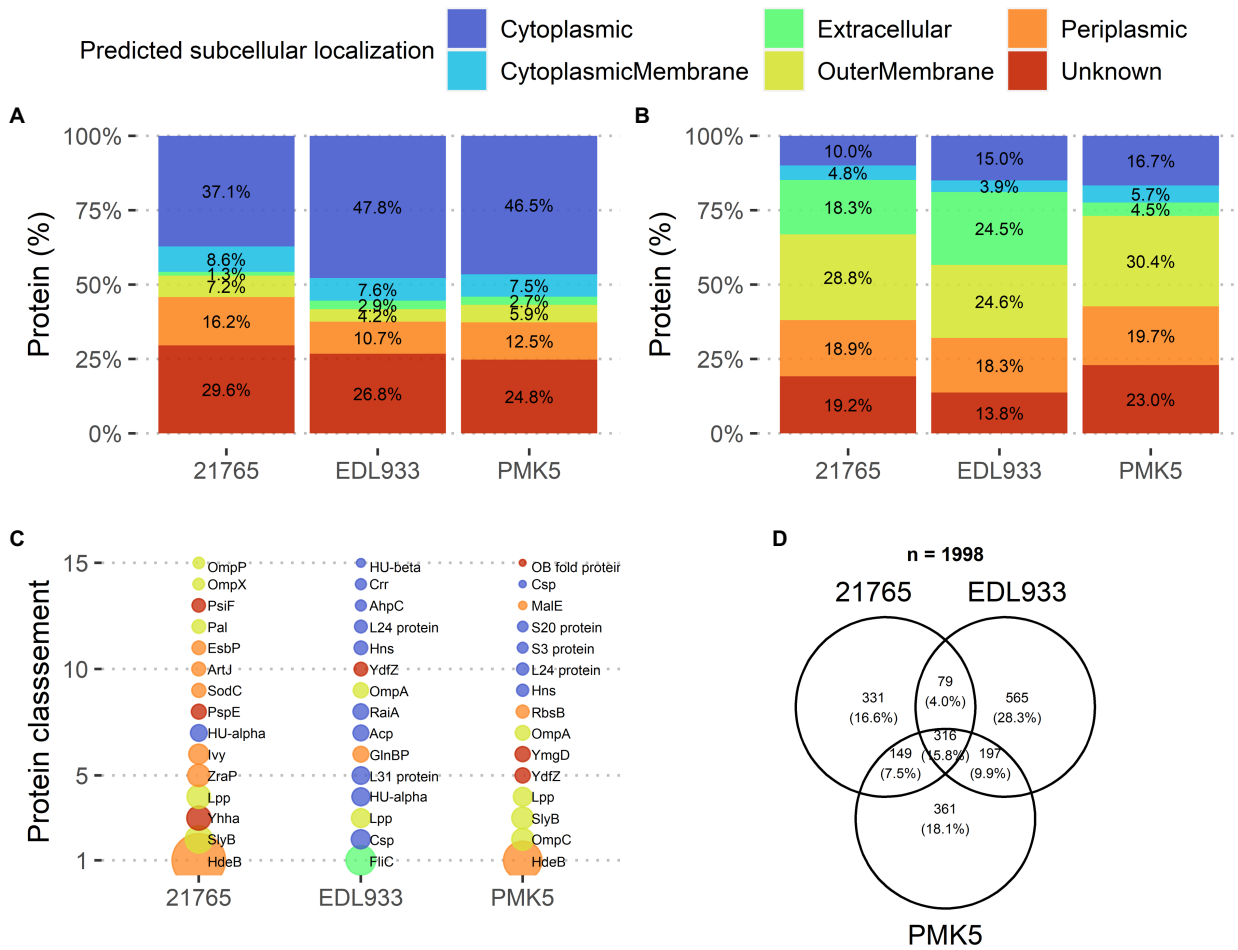
Qualitative description of surface protein extracts of STEC O26:H11 str. 21,765, O157:H7 str. EDL933, and O103:H2 str. PMK5, analyzed by MS/MS. **(A)** Distribution of identified proteins based on subcellular localization prediction for each strain (top left). **(B)** Distribution of identified proteins for each strain according to the relative protein abundance (NSAF) normalized to the number of identified proteins in the corresponding subcellular localization group (top right). **(C)** Ranking of the 15 predominant proteins identified in protein extracts for each strain according to relative abundance (NSAF) (bottom left). The bubble size is proportional to the relative abundance value. **(D)** Venn diagram of the identified proteins according to the strain (bottom right). Acp, acyl carrier protein; AhpC, peroxiredoxin; ArtJ, arginine ABC transporter substrate-binding protein; Crr, Glucose-specific enzyme IIA component of PTS; Csp, cold shock-like protein; EsbP, extracellular solute-binding protein 3; FliC, flagellin; GlnBP, glutamine-binding periplasmic protein (GlnBP); HdeB, acid stress chaperone HdeB; Hns, DNA-binding protein; HU-alpha, DNA-binding protein HU-alpha; HU-beta, DNA-binding protein HU-beta; Ivy, C-lysozyme inhibitor; L24 protein, 50S ribosomal protein L24; L31 protein, 50S ribosomal protein L31; Lpp, major outer membrane lipoprotein Lpp; MalE, maltodextrin-binding protein; OB fold protein, bacterial OB fold family protein; OmpA, outer membrane protein A; OmpC, gram-negative porin; OmpP, omptin family outer membrane protease OmpP; OmpX, outer membrane protein X; Pal, peptidoglycan-associated protein; PsiF, phosphate starvation-inducible protein; PspE, phage shock protein E; RaiA, ribosome-associated translation inhibitor RaiA; RbsB, D-ribose transporter subunit RbsB; S20 protein, 30S ribosomal protein S20; S3 protein, 30S ribosomal protein S5; SlyB, outer membrane lipoprotein SlyB; SodC, superoxide dismutase; YdfZ, putative selenoprotein YdfZ; Yhha, uncharacterized protein YhhA; YmgD, YmgD family protein; ZraP, zinc resistance-associated protein.

A total of 1,157, 875, and 1,023 *E. coli* proteins were identified in STEC protein extracts from the O157:H7 EDL933, O26:H11 21,765, and O103:H2 PMK5 strains, respectively (Supplementary Tables 1–3). These proteins extracts were qualitatively composed primarily of proteins assigned to a cytoplasmic (37.1–47.8%), unknown (24.8–29.6%), or outer membrane (16.2–10.7%) localization (Figure 4A). Analysis of the relative abundance of each predicted localization showed a high percentage of proteins that were localized at the outer membrane (30.3–18.3%) or were secreted (41.55–9.4%) (Figure 4B). Interestingly, the amount of protein expected to be extracellular was lower for the 21,765 strain than for the other two strains. The quantitative proteomic approach showed that many cytoplasmic proteins were present in the STEC protein extracts in small quantities, while fewer membrane proteins were present, but in larger quantities. Figure 4C shows the top 15 proteins in the extracts. The most abundant proteins in the extracts were predicted to be extracellular for the EDL933 strain (flagellin) and periplasmic for the 21,765 and PMK5 strains (acid stress chaperone HdeB). The relative abundance of the acid stress chaperone HdeB of the both strains was higher than the remaining proteins as indicated by the larger diameter of the bubble in Figure 4C. In the PMK5 strain, three outer membrane proteins were in the top 5 (the outer membrane porin C, the outer membrane lipoprotein SlyB and the major outer membrane lipoprotein Lpp). The most abundance proteins appeared to be similar for the 21765 and PMK5 strains. For the EDL933 strain, the abundance seemed more homogenous. Regardless of the strain, 1,998 unique *E. coli* proteins were identified and among them, 316 (15.8%) were shared between the three strains (Figure 4D). EDL933 had the highest number of unique proteins (565 proteins, 28.3%), while 331 (16.6%) and 361 (18.1%) proteins were unique to the 21765 and PMK5 strains, respectively.

#### Relative tandem mass tag quantitative proteomics

**Figure 5:**
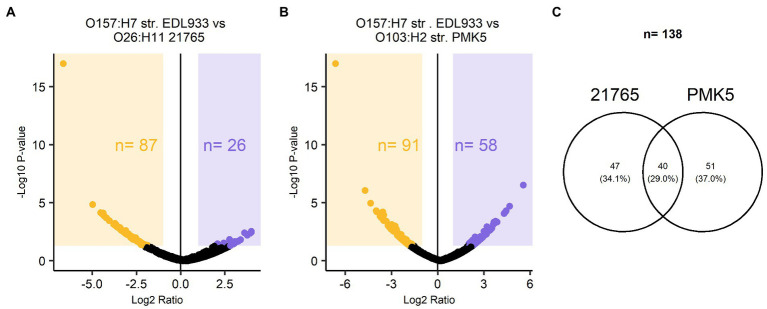
Relative quantitative description of TMT-labelled surface protein extracts of STEC O26:H11 str. 21765, O157:H7 str. EDL933, O103:H2 str. PMK5. Volcano plot of protein abundance ratios and the associated *p*-values for the pairwise comparisons **(A)** EDL933 vs. 21765 and **(B)** EDL933 vs. PMK5. Purple and orange panels correspond to proteins with at least a two-fold change in expression. The purple panel marks the differentially expressed proteins (DEPs) of the EDL933 strain, while the orange panel highlights those of the 21765 and PMK5 strains for **(A,B)**, respectively. **(C)** Venn diagram of proteins with at least a two-fold change in expression in the MFGMP-adhering strains, O26:H11 str. 21765 and O103:H2 str. PMK5.

**Figure 6:**
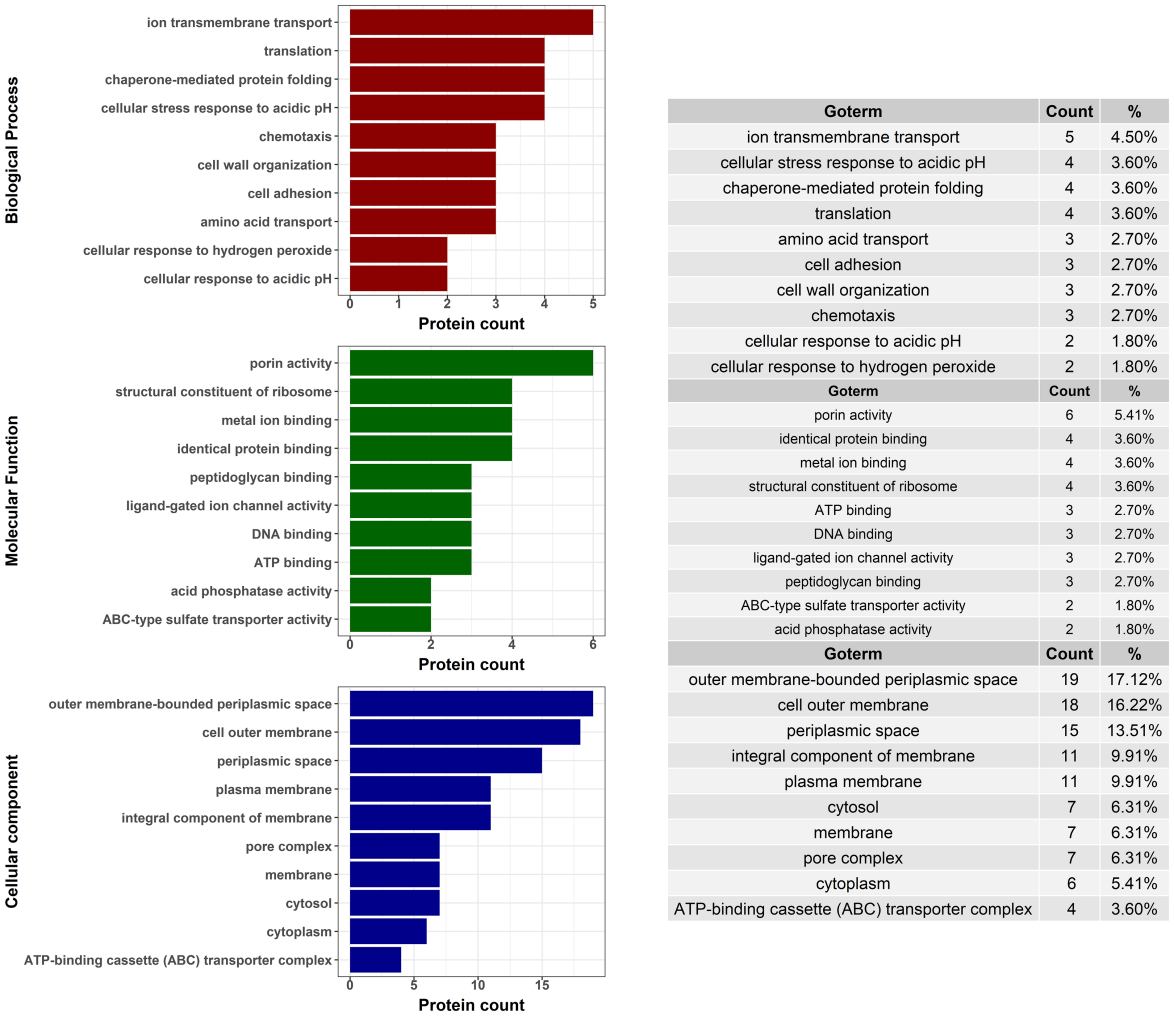
Gene ontology (GO) analysis of proteins with at least a two-fold change in expression for STEC strains that exhibited MFGMP adhesion capabilities, O26:H11 str. 21765 and O103:H2 str. PMK5.

TMT-labeled sample analysis found 1,192 proteins that could be used for relative quantitive assessment (Supplementary Table 4). We found 87 and 91 proteins (a total of 178 proteins) that were expressed at least two-fold higher in the 21,765 and PMK5 strains than in the EDL933 strain (Figures 5A,B). Among these 178 proteins, 40 proteins were present in both the 21,765 and PMK5 strains (Figure 5C) and 25 proteins were uncharacterized proteins for which no information was available even from homologous proteins (Supplementary Table 5). Consequently, 113 proteins were used for Gene Ontology (GO) term analysis to identify their subcellular localization and/or biological function (Figure 6). More proteins were associated with cellular component than with molecular function or biological process. The top 3 GO terms were “outer membrane-bounded periplasmic space” (17.12%), “cell outer membrane” (16.22%), and “periplasmic space” (13.51%). Concerning biological processes, “cell adhesion” was counted three times behind “ion transmembrane transport” (5), “cellular stress response to acidic pH,” and “chaperone-mediated protein folding” (4). In total, 17 proteins were associated with the biological processes “cell adhesion and/or the cellular component” or “cell outer membrane” and not localized in the inner leaflet of the OM or the periplasmic space (Table 1). Several outer membrane proteins (OmpC, OmpN, OmpP, NmpC, intimin, AIDA-I, maltoporin, LptD) and lipoproteins (Blc, LptE, LptB, TraT, BhsA) were more highly expressed in 21,765 or PMK5 strains than in the EDL933 strain. Five proteins (OmpN, OmpC, Blc, LptE) were expressed by both adhering strains (21,765 and PMK5) with an approximatively equivalent fold change (FC) compared with the EDL933 strain (from 4.26 to 9.58, Table 3). The differentially expressed proteins (DEPs) had a FC between 2 and < 10, except the autotransporter AIDA-I expressed by the 21,765 strain and the porin expressed by the PMK5 strain, which had FCs of approximately 23 and 13, respectively, compared with EDL933.

**Table 3:** Surface proteins of STEC O26:H11 21,765 and O103:H2 PMK5 strains that could be involved in MFGMP adhesion.

Strain	Protein	Accession (homologous)	Gene ontology (GO)	Fold change of 21,765 compared with EDL933 (*p*-value)	Fold change of PMK5 compared With EDL933 (*p* -value)	Fold change of 21,765 compared with PMK5 (*p* -value)
21765 and PMK5	Outer membrane protein N	F4NE19	Cell outer membrane [GO:0009279]; pore complex [GO:0046930]; porin activity [GO:0015288]; ion transmembrane transport [GO:0034220]	4.99 (0.0282)	5.78 (0.0082)	1.14 (0.8560)
21765 and PMK5	Outer membrane lipoprotein Blc	A0A7U9G1P7	Cell outer membrane [GO:0009279]; lipid binding [GO:0008289]	5.90 (0.0172)	4.06 (0.0311)	1.17 (0.6747)
21765 and PMK5	Outer membrane pore protein C	D6IC31	Cell outer membrane [GO:0009279]; pore complex [GO:0046930]; porin activity [GO:0015288]; ion transmembrane transport [GO:0034220]	8.94 (0.0040)	9.00 (0.0011)	1.17 (0.8095)
21765 and PMK5	LPS-assembly lipoprotein LptE	W8T1H6	Cell outer membrane [GO:0009279]; Gram-negative-bacterium-type cell outer membrane assembly [GO:0043165]	9.58 (0.0031)	3.89 (0.0314)	2.60 (0.0388)
21765 and PMK5	LPS-assembly protein LptD	A0A0K3M6Z5 (P31554)	Cell outer membrane [GO:0009279]; transporter complex [GO:1990351]; Gram-negative-bacterium-type cell outer membrane assembly [GO:0043165]; lipopolysaccharide export [GO:0015921]; response to organic substance [GO:0010033]	6.15 (0.0129)	4.26 (0.0147)	1.49 (0.3188)
21765	Outer membrane porin protein (fragment)	K4Y655	Cell outer membrane [GO:0009279]; pore complex [GO:0046930]; porin activity [GO:0015288]; ion transmembrane transport [GO:0034220]	8.22 (0.0055)	1.60 (0.3668)	5.66 (0.0002)
21765	Omptin family outer membrane protease (OmpP)	A0A5D8M8W6	Cell outer membrane [GO:0009279]; integral component of membrane [GO:0016021]; aspartic-type endopeptidase activity [GO:0004190]	8.28 (0.0054)	1.16 (0.6792)	6.96 (0.0000)
21765	Autotransporter adhesin AIDA-I (Diffuse adherence adhesin)	F8SKE5	Cell outer membrane [GO:0009279]; cell surface [GO:0009986]; extracellular region [GO:0005576]; integral component of membrane [GO:0016021]; periplasmic space [GO:0042597]	22.94 (0.0001)	1.47 (0.7208)	34.06 (0.0000)
21765	Intimin (Attaching and effacing protein) (Eae protein)	A0A3Z7VG60 (Q07591)	Cell outer membrane [GO:0009279]; integral component of membrane [GO:0016021]; cell adhesion [GO:0007155]	8.34 (0.0053)	1.11 (0.7209)	7.11 (0.0000)
PMK5	Intimin (Attaching and effacing protein) (Eae protein)	H3JV43	Cell outer membrane [GO:0009279]; integral component of membrane [GO:0016021]; cell adhesion [GO:0007155]	1.49 (0.4368)	6.11 (0.0065)	4.92 (0.0013)
PMK5	Outer membrane porin protein NmpC	D6I5J3	Cell outer membrane [GO:0009279]; pore complex [GO:0046930]; porin activity [GO:0015288]; ion transmembrane transport [GO:0034220]	1.12 (0.6560)	6.23 (0.0059)	5.62 (0.0005)
PMK5	TraT complement resistance protein	D9Z4U4	Cell outer membrane [GO:0009279]; conjugation [GO:0000746]; regulation of conjugation [GO:0046999]	1.29 (0.6466)	9.38 (0.0009)	8.34 (0.0000)
PMK5	Gram-negative porin	D7Y4X5	Cell outer membrane [GO:0009279]; pore complex [GO:0046930]; porin activity [GO:0015288]; ion transmembrane transport [GO:0034220]	1.39 (0.6251)	13.00 (0.0002)	8.63 (0.0000)
PMK5	Multiple stress resistance protein BhsA (Copper-induced outer membrane component)	A0A070FQT8 (P0AB40)	Cell outer membrane [GO:0009279]; response to copper ion [GO:0046688]	3.27 (0.0949)	3.01 (0.0451)	1.09 (0.7891)
PMK5	Vitamin B12 transporter BtuB (Cobalamin receptor) (Outer membrane cobalamin translocator)	A0A0E1LF30 (A8A774)	Cell outer membrane [GO:0009279]; pore complex [GO:0046930]; ABC-type vitamin B12 transporter activity [GO:0015420]; metal ion binding [GO:0046872]; porin activity [GO:0015288]; ion transport [GO:0006811]	2.43 (0.1598)	3.78 (0.0395)	1.27 (0.6785)
PMK5	Maltoporin LamB	A0A070V5G2 (A7ZUQ8)	Cell outer membrane [GO:0009279]; pore complex [GO:0046930]; maltodextrin transmembrane transporter activity [GO:0042958]; maltose transporting porin activity [GO:0015481]; ion transport [GO:0006811]	1.14 (0.6401)	5.03 (0.0141)	4.69 (0.0019)
PMK5	Major type 1 subunit fimbrin (Pilin)	A0A0H2V3I0	Pilus [GO:0009289]; cell adhesion [GO:0007155]	1.88 (0.3636)	3.53 (0.0295)	2.03 (0.1283)

Fold change (FC) was calculated by comparing the protein abundance in the 21,765 and PMK5 strains with that in the EDL933 strain, which showed low adhesion to MFGMPs. For each strain, three independent protein extracts were analyzed to perform statistical analysis (*t*-test and the associated value of *p*).

## Discussion

Shiga toxin-producing *E. coli* (STEC) may cause severe human infections mainly through ingestion of contaminated food, including raw milk products. The milk fat globule membrane (MFGM), which surrounds MFGs, is well known for its anti-adhesive proprieties against pathogenic bacteria, including STEC (Ofek et al., 2003; Claeys et al., 2013; Yoon et al., 2016; Douëllou et al., 2018; Bagel and Sergentet, 2022). However, the mechanisms involved are unknown. We previously demonstrated that the affinity of STEC for the enriched-MFG layer was serotype-dependent for the strains belonging to the O157:H7 serotype and strain-dependent for O26:H11 and O103:H2 strains (Bagel et al., 2022). Published literature suggests that (glyco)-proteins anchored to the MFGM may serve as potential ligands for bacteria, as well as for STEC adhesins, and thus act as mimetic receptors (Douëllou et al., 2017; Bagel and Sergentet, 2022). Some knowledge gaps exist regarding STEC adhesion strategies, especially for the non-O157 STEC strains (O26:H11 and O103:H2 strains) that are largely less studied than O157:H7 strains (Koutsoumanis et al., 2020). It is likely that STEC uses strain- and/or serotype-specific adhesion mechanisms to invade hosts. The nature and type of proteins exposed at the STEC surface could explain the strain and/or serotype effect in STEC’s affinity for MFGs that we highlighted previously (Bagel et al., 2022). In this study, we used phenotypic assays to evaluate the capacity of STEC strains to form molecular interactions with bovine milk fat globule membrane proteins (MFGMPs) and proteomics to highlight adhesion factors potentially involved in this phenomenon.

We evaluated the major non-specific interactions involved in bacterial adhesion (hydrophobic and surface charge). At the collection level, STEC and AEEC strains were mainly hydrophilic, except for three strains belonging to the O103:H2 serotype (Figure 2). Hamadi et al. (2008) showed by water contact angle that *E. coli* strains are hydrophilic. However, the *E. coli* O157:H7 strain has also been described as hydrophobic (Patel et al., 2011). Witsø et al. (2014) reported a cell surface hydrophobicity of between 50 and 70% for *E. coli* O103:H2 strains, consistent with our results. Bacterial hydrophobic forces are influenced by the nature of the membrane-anchored components, including amino residues that are exposed to the extracellular environment (Law, 2015). MFGs are highly hydrophobic in their central region (hydrophobic TAG core) but the MFGM, rich in polar lipids and glycoconjugates, allows MFGs to float in the aqueous phase of milk (Corredig and Dalgleish, 1998; Chevalier and Sommerer, 2011). Globally, MFGs and STEC are both dispersed in the aqueous phase of milk due to the polar properties of the components anchored in their membrane (proteins, polar lipids). Hydrophobic forces are also influenced by physicochemical conditions such as temperature, A_w_, ionic strength, and pH (Hamadi et al., 2004). Moreover, several parameters intrinsic to the MATH test such as vortex time, hydrophilic phase/hydrophobic phase ratio, and solvent type, can influence results (Nachtigall et al., 2019). Similarly, culture conditions directly affect the formation of hydrophobic bonds mainly by modulating surface protein expression.

We found that the surface charges of MFGs and the AEEC strains in the real aqueous phase of raw milk were both negative and of similar orders of magnitude: −12.2 ± 0.14 to −10.7 ± 0.6 mV and − 3.7 ± to −2.9 mV, respectively. Our results are similar to published data showing that the surface charge of raw MFGs is between −12.2 and − 9 mV (Lopez et al., 2007; Obeid et al., 2019; Verma et al., 2019). The bacterial cell surface is generally negatively charged because of the carboxyl and phosphate core as well as the lipopolysaccharide (LPS) located at the surface (Hong and Brown, 2008). The surface charge of *E. coli* is reported to be negative (Kłodzińska et al., 2010). Furthermore, Liang et al. (2016) showed a large range in ζ-potential that varies according to *E. coli* strain (−6.8 to −39.9 mV). ζ-potential measurements were made at pH 6.7 in presence of milk minerals that screen negative charges. In a previous study, we showed that AEEC strains have different affinities for the raw milk layer (Bagel et al., 2022), suggesting that bacterial surface charges may not be the main factor influencing STEC-MFG association. It appears that non-specific interactions are not strongly involved in opposing electrostatic forces and that, instead, specific/molecular interactions may be implicated. Interestingly, we have shown that the O103:H2 str. 2503, str. 32396, and str. 445–14 display a similar affinity for the raw milk cream layer and these strains have a hydrophobic profile (Bagel et al., 2022). However, there are other non-specific interactions involved in bacterial adhesion to a surface, such as Lewis and Van Der Waals bonds (van Loosdrecht et al., 1990; Chen et al., 2014). Non-specific interactions alone cannot explain STEC’s affinity for MFGs. These results suggest that other adhesion mechanisms such as specific interactions must be involved in STEC’s affinity for MFGs.

Here we have demonstrated, for the first time to our knowledge, STEC’s capacity to adhere to MFGMPs and we have highlighted a serotype effect. For 10 of the 16 assessed strains, the ΔTt increased as a function of the MFGMP concentration (Figure 3), suggesting an adhesion to these ligands. Among the strains analyzed, all O103:H2 strains, four O26:H11 strains, and the K-12 MG1655 strains seemed capable of adhering to MFGMPs. However, the strains belonging to the O157:H7 serotype (n = 5) and O26:H11 str. 4,315-A adhered very weakly to MFGMPs in our experimental conditions. Moreover, we found that the presence of the *stx* gene (STEC vs. AEEC) and the strain origin (Table 1) did not seem to be correlated with adhesion capacity. We obtained similar results across independent experimental replicates, supporting the hypothesis that STEC-MFGMP adhesion is specific rather than non-specific. However, our results are quite surprising compared with the results of previously published experiments. We previously found that STEC strains belonging to the O157:H7 serotype have a higher capacity than O26:H11 and O103:H2 serotypes to concentrate in the cream of raw milk, which is rich in MFGs (Douëllou et al., 2018; Bagel et al., 2022). We assumed that a high concentration capacity in the cream was correlated with high capacity to adhere to MFGMPs. In our plate-washing experiment, it may be possible that the interaction of the O157:H7 strains with MFGMPs was weaker than the other strains or that the molecular targets of the O157:H7 strains may have been absent from the MFGM protein extract, denatured during the extraction process, or did not coat the wells. Furthermore, the mechanism underlying the concentration of STEC in the cream layer during natural creaming may be related to a completely different mechanism.

MFGMPs are membrane-bound proteins with both a hydrophilic region that is exposed to an aqueous environment at the surface of MFGs and a hydrophobic region anchored in the polar lipid trilayer in the membrane. Protein structure can be affected during extraction, leading to conformations that are different to the native conformation in the MFGM, which can hide the epitopes recognized by STEC for adhesion. Moreover, free MFGMPs may clump together, preventing the access of the epitopes to STEC. In addition to what has been said, the hydrophobic domains of the MFGMPs are not accessible to bacteria when they encounter intact MFGs, whereas in our trial these domains may have served as an anchoring point. To improve this model, we intend to work directly on MFGMPs in their original state, e.i. anchored in a biological membrane.

In addition, negative ΔTt values were observed for some strains, in particular the strains belonging to the O157:H7 serotype. A negative value corresponds to a loss of cells and could suggest that MFGMPs affect the viability of STEC strains. MFGMPs, especially xanthine oxidoreductase, have anti-bacterial properties (Martin et al., 2004). However, the bacteriostatic or bacteriocidal effect of MFGMPs in our experimental conditions was very weak. We previously evaluated the impact of MFGMPs on STEC growth and found that the presence of MFGMPs did not affect cell viability and did not modulate strain growth (data not shown). Therefore, the negative ΔTt values in this study seem to be more related to the sensitivity of the measurement method than to a biological phenomenon (the zero was included in the standard deviation), except for the O157:H7 str. 2,054 which had consistently negative values in all experiments.

In the second part of this work, we aimed to identify STEC surface proteins that were preferentially expressed by O26:H11 str. 21,765 and O103:H2 str. PMK5 and, therefore, represented an adhesion profile for MFGM. Previous studies have shown that the presence of MFGs can modulate adhesion of bacterial cells, including STEC (Ross et al., 2016; Douëllou et al., 2018). We can therefore assume that the STEC proteins involved in the recognition of eukaryotic cells play a role in MFG adhesion. Studies on STEC diversity have shown a clustering of strains according to serotype. Furthermore, strains belonging to O26:H11 and O103:H2 serotypes were grouped into subgroups. In this study, we found that more proteins were specific to the O157:H7 str. EDL933 than the two other strains analyzed (Figure 4D). It is possible that the O157:H7 str. EDL933, which is genetically different from the other strains of the collection, did not have or did not express adhesion factors that recognize MFGMPs. The surface protein extracts from the O157:H7 str. EDL933, O26:H11 str. 21,765, and O103:H3 str. PMK5 were characterized by TMT-nLC-MS/MS to determine cell surface components that may influence the adhesion of STEC to MFGMPs. Quantitative proteomic analysis showed that the O26:H11 str. 21,765 and O103:H3 str. PMK5 differentially expressed five outer membrane-associated proteins or lipoproteins, compared with the EDL933 strain (Table 3). This analysis also showed strain-specific differentially expressed proteins (DEPs) that could be involved in a strain-dependent adhesion mechanism. In the O26:H11 21,765 strain, four proteins or glycolipids were differentially expressed, while eight DEPs were found in the PMK5 strain.

Several outer membrane proteins (OMPs) involved in STEC eukaryotic cell adhesion were identified in both the 21,765 and PMK5 extracts. The outer membrane proteins C (OmpC) and N (OmpN), common to the 21,765 and PMK5 strains, are both porins allowing passive diffusion of small molecules across the outer membrane (OM). OmpN has been less well characterized in the literature, but has a high homology to OmpF and OmpC (Prilipov et al., 1998; Dam et al., 2017). We also identified two other membrane proteins in 21,765: (1) the outer membrane protein P (OmpP) is an outer membrane protease that belongs to the omptin family and protects cells from cationic antimicrobial peptides and inflammatory responses and promotes adherence to eukaryotic cells (Hritonenko and Stathopoulos, 2007; Hwang et al., 2007) and (2) a putative porin that has 50% homology with the YedS porin of the *E. coli* K-12 MG1655 strain. In the PMK5 strain, the porin NmpC, homologous to OmpC and OmpF (Prilipov et al., 1998), was detected, as well as an OMP homologous to OmpC and associated with the accession number D7Y4X5. NmpC is not expressed in the K-12 MG1655 strain (Blasband et al., 1986), which displayed an adhesion profile. Several porins similar to OmpC have been identified.

*OmpC* deletion decreases the adhesion and invasion capacities of *E. coli* for intestinal epithelial cells (Rolhion et al., 2007). Furthermore, OmpC binds lactoferrin, a glycoprotein found in milk (Sallmann et al., 1999). Lactoferrin is a minor protein of MFGMs (Lee et al., 2018). Therefore, OmpC, and perhaps homologous porins, may bind lactoferrin in MFGMs. The OmpA encoded by some *E. coli* strains has affinity for the GlcNAc-β-(1,4)-GlcNAc epitopes that comprise the core of all N-linked glycans (Prasadarao et al., 1996). N-glycosites, including mucins (MUC15 and MUC1), butyrophilin (BTN), lactadherin (LDH) and cluster of differentiation 36 (CD36), have been found on the extracellular domain of bovine MFGMPs (O’Riordan et al., 2014). Mannose residues are present on BTN and CD36 (O’Riordan et al., 2014). We also identified another porin, the maltoporin LamB, involved in the transport of maltose and maltodextrins, in the PMK5 strain. LamB is an alternative or additional adherence factor for EPEC and acts as a receptor for several bacteriophages (Randall-Hazelbauer and Schwartz, 1973; Subramanian et al., 2008). The PMK5 strain also expressed the OMP BhsA, which lowers the permeability of the outer membrane to copper and modulates biofilm formation by decreasing cell aggregation and cell surface adhesion (Zhang et al., 2007; Mermod et al., 2012).

The major type 1 subunit fimbrin (FimA) that constitutes the pilus rod of the Type 1 Fimbriae (T1P), was also identified in the PMK5 strain. While the *fimA* gene sequence is highly variable between different *E.coli* strains (Kisiela et al., 2013), FimA expression was not significantly different in PMK5 compared with 21,765, suggesting that this strain also expresses a signficant amount of the protein (Table 3). The key adhesin component in T1P is carried by FimH which can bind to the mannose residues of some receptors on eukaryotic cells (Ageorges et al., 2020). We identifed FimH in the three protein extracts but the expression ratio was not significantly different (Table 3). *E. coli* T1F can bind to glycoprotein 2 (zymogen granule membrane), a minor protein anchored at the MFGM (Murgiano et al., 2009). In addition, a study by Shaikh et al. (2007) found an N135K mutation in FimH of *E. coli* O157:H7 strains belonging to the cluster 2 and 3, including the EDL933 and SAKAI strains, that impaired the affinity for mannose. A decrease in mannose adhesion capabilities of O157:H7 strains, but not O103:H2 and O26:H11 strains, which have an intact FimH protein, could help to explain our results.

In addition, the β-subtype of intimin, which is responsible for attaching and effacing lesions, was identified in the 21,765 strain, while the ε-subtype was identified in the PMK5 strain (Table 3). These two intimin variants are different from the γ-subtype variant identified in the EDL933 strain (Supplementary Table 1). Intimin is the main eukaryotic cell adhesion factor of STEC, forming a very specific adhesion bond with Tir (Translocated intimin receptor), as well as with eukaryotic proteins nucleolin and β1 integrin (Sinclair et al., 2006). The MFGM expresses low levels of integrins but has not been shown to express nucleolin (Bagel and Sergentet, 2022). The 21,765 strain also expresses the AIDA-I autotransporter which acts as a bacterial adhesin mediating attachment to a broad variety of mammalian cells and facilitating bacterial autoaggregation (Vo et al., 2017). AIDA-I is also a highly efficient initiator of biofilm formation (Sherlock et al., 2004) and is involved in cellular adhesion of diarrheagenic *E. coli (DEC)* (Ageorges et al., 2020).

The 21,765 and PMK5 strains also differentially expressed some outer membrane lipoproteins (Blc and the LptD-LptE complex). The lipoprotein Blc belongs to the lipicalin family, an important class of lipid transfer proteins that are anchored in the inner leaflet of the outer membrane and bind to fatty acids or phospholipids (Campanacci et al., 2004, 2006). LPS-assembly protein LptD and lipoprotein LptE form a complex at the OM surface, allowing transport of LPS at the cell surface (Sperandeo and Polissi, 2016). We do not have sufficient information to conclude whether these proteins are directly involved in bacterial and MFGMP adhesion. The O-antigen, which is part of LPS, is a highly variable region that is used to classify bacteria based on sugar composition (Liu et al., 2020). In addition, the flagellar H antigen (*fliC*), which is used as a complement to O-antigen for classification, is known to be involved in adhesion. Furthermore, the FliC protein, specific to each strain assayed, composed the majority of the protein extract of the EDL933 and PMK5 strains (Supplementary Tables 1, 2). Therefore, FliC_H2_ of the PMK5 strain could be involved in MFGMP adhesion. PMK5 also expressed: (1) the vitamin B12 transporter BtuB, involved in the active translocation of vitamin B12 across the outer membrane to the periplasmic space and (2) the TraT protein, an OM lipoprotein that prevents unproductive conjugation between bacteria carrying similar plasmids. There is no published evidence that these lipoproteins are involved in bacterial adhesion althought BtuB was described as a phage receptor (Li et al., 2019).

Of the 178 DEPs highlighted by our study, 25 DEPs were not included in our further analysis due to gaps in available information (Supplementary Table 5). Further characterization of these proteins could highlight novel protein candidates. In addition, we found that protein extracts were mainly enriched in proteins associated with the outer membrane or the extracellular component (Figure 4B). However, we also identified a large proportion of periplasmic proteins, reflecting leakage during the extraction protocol of the periplasmic content. In contrast, few cytoplasmic membrane proteins were identified although cytoplasmic proteins were identified. These proteins may have originated from dead cells remaining in the culture medium or have leaked from the inner membrane. Some external membrane proteins could not be correctly extracted and therefore are absent from the analysis. We can also point out that some proteins that are more sensitive to heat than others might not be included in the extract. Therefore, these proteins, potentially involved in the adhesion process, are not covered by the study. Future studies using a more global approach may identify more proteins involved in this adhesion phenomenon.

In this study, we fixed the temperature of the STEC-MFGMP adhesion assay to 4°C to evaluate the adhesion of STEC to MFGMPs in milk storage conditions. Although contamination of raw milk with STEC mainly occurs during milking when the milk temperature reaches 37°C, milk is rapidly refrigerated to 4°C to assure its conservation (Quigley et al., 2013; Machado et al., 2017; Skeie et al., 2019). To exert their anti-adhesive properties against STEC, the STEC-MFG association must be possible in the environmental conditions of the human intestinal system. Therefore, the results observed here do not indicate that *E. coli* O157:H7 strains do not possess MFG adhesion capacity. As physicochemical parameters govern adhesion steps, further analysis of the effect of other parameters, such as pH, ionic strength, and temperature could help to understand the low adhesion of *E. coli* O157:H7 strains to MFGMPs observed in this study. The impact of these environmental factors on STEC-MFG association, as well as the presence of microbiota, should be evaluated to ensure that STEC-MFGMP interactions are achievable in the human GI tract. The adaptation of STEC to environmental factors could also explain the lower prevalence of STEC O157:H7 strains in raw milk products than STEC O26:H11 and O103:H2 strains (EFSA and ECDC (European Food Safety Authority and European Centre for Disease Prevention and Control), 2021). The serotype-dependent adhesion observed in this study shows the same pattern as the prevalence data. Bacteria belonging to the O26:H11 and O103:H2 serotypes, which seem to be more adapted to survival in milk and cheese (Miszczycha et al., 2013), show adhesion to MFGMPs. O157:H7 serotype bacteria grow more slowly than O26:H11, O103:H2, and O145:H28 serotypes. These data suggest that O26:H11, O103:H2, and O145:H28 strains may be better adapted to the environment (physicochemical parameters and microbiota) found in cheeses. The ability of STEC cells to adhere to MFGs could be considered to be an advantageous mechanism.

The use of antibiotics to treat STEC infection is particularly contentious due to their impact on the expression of Shiga toxin genes. In the field of health and nutrition, raw milk fat is gaining popularity due to the richness and variety of proteins linked to its membrane. MFGMP’s anti-adhesive abilities against pathogenic bacteria, including STEC, might be a promising prophylactic and alternative approach to anti-infection treatments. As bacterial adhesion is the first step of infection, inhibiting this step is a key strategy for infection control. Competition for the natural binding sites of pathogenic bacteria by mimetic receptors could inhibit pathogen attachment. Such strategies have already been described for other enteric pathogens (Asadi et al., 2019). The mechanisms of adhesion between STEC and MFGM should be further studied to uncover new therapeutic solutions. Here, we highlight some outer membrane proteins or lipoproteins that may be involved in the adhesion of STEC to MFGs but further studies are needed. It will be essential to validate the involvement of STEC candidate proteins in adhesion to MFGs and intestinal cells and to further identify their ligands among MFGMPs.

## Conclusion

In this study, adhesion of Shiga toxin-producing *E.coli* (STEC) to milk fat globule membrane proteins (MFGMPs) was observed for the first time and a serotype effect was discovered. The strains belonging to the O157:H7 serotype showed no adhesion to MFGMPs, whereas the strains belonging to the O26:H11 and O103:H2 serotypes appeared to adhere to MFGMPs. We also showed that non-specific interactions, hydrophobic forces, and surface charges do not seem to be major forces involved in STEC-MFG association. We identified several outer membrane proteins—OmpC and homologous proteins, intimin, Type 1 Fimbriae, and AIDA-I—that may be involved in STEC-MFG adhesion. More work is needed to elucidate the level of involvement of these proteins. As stated in the published literature, bacteria-MFGMP adhesion is a complex and multifactorial mechanism, likely involving several different strategies depending on the strain and/or serotype. The adhesion capabilities and the strength of STEC-MFGMP association needs to be further studied in various biological environments, such as raw milk cheese products and the human gastrointestinal tract, to follow the evolution of the STEC-MFG complex and their anti-adhesion properties to intestinal epithelium.

## Data availability statement

The data presented in the study are deposited in the MassIVE repository, accession number MSV000089913.

## Author contributions

AB, CL, VM, TD, and DS contributed to conception and design of the study. AB, ED-B, and CL performed the experiments. AB realized data analysis and visualization. AB and DS wrote the first draft of the manuscript. CL, VM, and TD reviewed and edited the work. All authors contributed to the article and approved the submitted version.

## Funding

This study was funded by the Centre National Interprofessionnel de l’Economie Laitière (CNIEL, French Dairy Interbranch Organization, Paris). The French Ministry of Agriculture (Paris, France) and VetAgro Sup (Marcy-L’Etoile, France) have also funded part of this work.

## Conflict of interest

The authors declare that the research was conducted in the absence of any commercial or financial relationships that could be construed as a potential conflict of interest.

## Publisher’s note

All claims expressed in this article are solely those of the authors and do not necessarily represent those of their affiliated organizations, or those of the publisher, the editors and the reviewers. Any product that may be evaluated in this article, or claim that may be made by its manufacturer, is not guaranteed or endorsed by the publisher.
